# Short-term clinical outcomes of patients admitted with chronic liver disease to selected teaching hospitals in Ethiopia

**DOI:** 10.1371/journal.pone.0221806

**Published:** 2019-08-30

**Authors:** Behailu Terefe Tesfaye, Esayas Kebede Gudina, Dula Dessalegn Bosho, Teshale Ayele Mega

**Affiliations:** 1 School of Pharmacy, Department of Clinical Pharmacy, Jimma University, Jimma, Ethiopia; 2 School of Medicine, Department of Internal Medicine, Jimma University, Jimma, Ethiopia; Azienda Ospedaliero Universitaria Careggi, ITALY

## Abstract

**Background:**

Chronic liver disease (CLD) is a progressive destruction of liver tissue with subsequent necrosis that persists for at least 6 months. In Ethiopia, despite the high burden report, data on CLD is limited. The objective of this study was to assess short-term clinical outcomes in patients admitted with chronic liver disease to three tertiary teaching hospitals in Ethiopia and to identify predictors of mortality.

**Methods:**

A cohort of 109 patients admitted with CLD to three tertiary teaching hospitals in Ethiopia, were prospectively followed from the time of admission to 30-days of hospital discharge. The study was conducted from April 1, 2018, to October 5, 2018. Kaplan-Maier curve was used to estimate survival and cox-regression analysis to identify predictors of mortality.

**Result:**

A total of 109 patients (80% male) diagnosed with CLD were included. Median age of the participants was 38(IQR, 30–48). The overall median length of hospital stay was 7(IQR, 4–11) days. Of the total, 39(35.8%) patients were HBsAg positive, and 12(11%) patients were anti-HCV positive. The 30-day mortality was 38(34.9%), and most of the deaths, 31(81.6%) occurred before hospital discharge. Hepatic encephalopathy at admission; being with unidentified risk factor/etiologies of CLD and total bilirubin level were independent predictors of in-hospital mortality. Patients with hepatic encephalopathy at admission had approximately 11 times increased risk of death as compared to patients without hepatic encephalopathy at admission. Similarly, the hazard of mortality was 5.8 times higher in those patients with unidentified risk factor/etiology as compared to others. The risk of dying had also increased with an increase in bilirubin (1.188[95% CI, 1.0719–1.316]) level.

**Conclusion:**

Approximately one-quarter of patients with CLD died during their hospital stay, and the risk of death continued after hospital discharge. Hepatic encephalopathy at admission, unidentified risk factor/etiology and increased level of total bilirubin are poor prognostic factors. Given that more than one third the patients had HBV-infection, access to antiviral drugs could help improve the prognosis of patients with end-stage liver disease in Ethiopia, as well as prevent the progression of the disease if initiated earlier.

## Introduction

Chronic liver disease (CLD) is a progressive destruction and regeneration of liver tissue with subsequent necrosis that persists for at least 6 months)[1]. Cirrhosis is the end spectrum of all CLD characterized by advanced fibrosis, scarring, and formation of regenerative nodules leading to hepatic architectural distortion[2]. It is characterized by the longest asymptomatic phase of compensated cirrhosis, followed by decompensated phase characterized by the occurrence of complications. The rate of transition is estimated to be 5%-7% per year and this period of transition is a critical step, which ends up in hepatic decompensation unless controlled[3,4].

There are several causes of CLD, among which, viral causes are recognized as a major public health challenge that requires an urgent response[5,6]. According to the 2017 Global Hepatitis Report of World Health Organization, viral hepatitis has caused 1.34 million deaths in 2015. Hepatitis B and C were responsible for 96% of the total mortality. Most of these deaths were due to CLD, particularly cirrhosis and hepatocellular carcinoma (HCC) accounted for 720000 and 470000 deaths, respectively[7].

CLD has significant public health and economic impact[8,9]. According to Global Burden of Disease study(GBD), CLD had caused an estimated 1,322,867.92 mortality (2.36% of the all-cause mortality), and 41,397,987.89 Disability-Adjusted Life Years (DALYs) in the year 2017[10]. In 2015, Centers for Disease Control and Prevention (CDC) reported estimated mortality of 40,326(1.5% of total deaths) attributed to CLD, making it to rank 12^th^ among the 15 leading causes of death [11].

In Sub-Saharan Africa, GBD reported an estimated figure of 157,558.69 deaths (2.11% of the all-cause mortality) due to CLD in 2017. In this year, in Ethiopia, an estimated 16,068.94deaths were attributable to CLD Of all-cause deaths attributed to CLD in Ethiopia, HBV, HCV, and alcohol accounted for 0.008%, 0.01% and 0.004% of total deaths, respectively[10]. However, data from Ethiopia are scares, and the burden of CLD is likely to be grossly underestimated. Only a handful of studies have been carried out in Ethiopia previously, reporting that CLD ranks among the top ten causes of death in the adult population, with large geographical variations within the country [12–15]. However, most of the previous studies were old, and retrospective.

## Materials, methods, and procedures

### Ethics approval and consent to participate

Written informed consent was obtained from all participants. The study protocol was ethically approved by Jimma University Medical Center Institutional Review Board committee with a reference number of IHRPGD/192/18. For those patients with Hepatic encephalopathy, written consent was obtained from caregivers.

### Study design and aim

This is a hospital-based longitudinal study. This study was mainly aimed to assess the baseline characteristics, to evaluate short-term mortality, and to identify predictors of in-hospital mortality in patients admitted with CLD to three selected specialized teaching hospitals in Ethiopia. The study was also aimed to identify risk factors/etiology of CLD, and incident in-hospital acute complications of CLD.

### Study settings

Ethiopia is a landlocked country in the Horn of Africa [16]. Based on the most recent United Nations projections, this country is a home for an estimated population of 107.53 million. This makes it the second-most populous country in Africa next to Nigeria and the 14^th^most populous country in the world. The capital city is Addis Ababa[17]. Despite its wealth in culture, the economic wealth of its citizens is poor with a Gross Domestic Product (GDP) per capita of US$ 861 in 2017[18]. The 2017 Ethiopia’s Human Development Index (HDI) value was 0.463 which put the country in the low human development category ranking 173 out of 189 countries and territories[19]. Infectious and communicable diseases account for about 60–80% of the health problems in the country; likewise, non-communicable diseases are thought to be rapidly increasing in this country[20]. The study was conducted in the medical wards of three tertiary teaching hospitals in Ethiopia: Jimma University Medical center (JUMC); Saint Paul’s Hospital Millennium Medical College (SPHMMC) and Hiwot Fana Specialized University Hospital (HFSUH). JUMC is located in Jimma town, Southwest Ethiopia and is about 346 km away from Addis Ababa. JUMC serves as a tertiary referral center for a catchment population of over 15,000,000 [21]. SPHMMC is located in Addis Ababa. This hospital has an inpatient capacity of more than 700 beds and sees an average of 1200 clients daily[22]. HFSUH is a tertiary referral hospital located in Harar town, east Ethiopia. This hospital serves as a referral hospital for the entire eastern part of the Ethiopia, including the Eastern Oromia region, Dire Dawa city, the Somali region, and the Harari regional state[23].

### Study participants

All CLD patients admitted to internal medicine wards of the three selected hospitals during the study period and who met the following inclusion criteria were considered: diagnosis of CLD, adult (age≥18years old)[23], and patients willing to give consent. Readmission, CLD patients admitted for problems(s) unrelated to CLD, and pregnant CLD patients were excluded.

### Data collection tool

Data collection tool (consists of components relevant for collecting data on socio-behavioral and clinical characteristics) was developed after reviewing relevant literature and active patient follow-up charts. Data were collected prospectively from active patient`s medical chart, patients and/or caregivers report. Data on socio-behavioral and clinical characteristics [laboratory findings; etiology; major presenting complaints; CLD complications and management(s) at admission, chronic comorbid illnesses)] were recorded at admission and on follow-up. The CAGE questionnaire was used to assess the clinical significance of alcohol use problem. The questionnaire consists of four questions to assesses whether the individual has ever felt the need to **cut down** on his/her drinking, if people have ever **annoyed** him/her by criticizing his /her drinking, if the person has ever felt **guilty** about drinking, and if the person has ever felt that s/he needed a drink first thing in the morning (**eye-opener**) ‘to steady nerves or to get rid of a hangover’. The questionnaire was interpreted to local languages (Amharic). The interpretation from English to the local language was undertaken by individuals fluent in their mother tongue for the local language and English and back-translated and checked for consistency. Then, pretest was done to check the validity of the tool.

### Patient assessment and follow-up

Patients were followed from hospital admission to 30-days of hospital discharge. Censoring of the participants was undertaken on their last day of contact. During the hospital stay, patients were interviewed by data collectors (clinicians). Information on the use of alcohol was obtained and categorized as never users (represented by `NO`) and past/current users (represented by `YES`). Patients with two or more "YES" score indicate the possibility of alcoholism and clinical significance of the alcohol use problem. In these patients alcohol was considered as a risk factor for CLD. Khat usage information was obtained and patients were categorized as no history of khat use `NO`, and past/current users ‘YES`.

The diagnosis of CLD was based on the presence of events suggestive of CLD such as ascites, splenomegaly, encephalopathy, variceal bleeding and/or ultrasound findings of an irregular liver surface and/or liver parenchyma heterogeneity. Risk factors/Etiological spectrums of CLD were defined as follows:

■ Chronic HBV was defined as a positive HBsAg test result
■ Chronic HCV was defined as a positive anti-HCV test result
■ Alcoholic was defined as CLD patients with a CAGE score of two or more
■ Hepatic schistosomiasis was defined as the presence ova of Schistosoma mansoni in stool and/or Ultrasound finding of hepatic schistosomiasis features such as Periportal fibrosis.
■ Autoimmune hepatitis was defined as positive anti-nuclear antibodies(ANA)
■ Unidentified etiology: refers to CLD Patients in whom no etiology was traced.

In the present study, CLD complication implies major complications and includes ascites, Spontaneous Bacterial Peritonitis, hepatic encephalopathy, variceal bleeding, hepatorenal syndrome[24] and HCC. Decompensation was defined by the development of clinically evident complications of portal hypertension (ascites, variceal hemorrhage, hepatic encephalopathy) or liver insufficiency (jaundice)[4].

At admission, Spontaneous bacterial peritonitis was diagnosed clinically (fever, presence of abdominal pain) and/or objectively when the ascitic fluid absolute neutrophil count was>250/μL. In-hospital incidence of Spontaneous bacterial peritonitis was diagnosed when the ascitic fluid absolute neutrophil count was>250/μL(in patients deemed free of Spontaneous bacterial peritonitis at admission). Physician`s clinical judgment and diagnosis was used for incident hepatic encephalopathy with a further request for ascertainment. Incident acute or overt gastrointestinal bleeding was diagnosed clinically by physician with/without endoscopic confirmation. Overt gastrointestinal bleeding is bleeding visible in the form of hematemesis, melena, or hematochezia[25]. In this study, in-hospital mortality, in-hospital acute CLD complication(s) and 30-day mortality post-discharge was considered as clinical outcomes. Thirty-day mortality implies cumulative mortality from hospital admission to 30-days of hospital discharge. In-hospital death was ascertained by the treating physician and from the discharge summary note. Patient-reported 30-day status was obtained from patients, caregivers and/or close friend`s report.

### Laboratory tests and imaging

Complete blood counts were performed using hematologic analyzers: XT-1800i (Sysmex, Japan), KX-21 N™ (Sysmex, Japan), and Cell-Dyn 1800® (Abbot, USA). Blood tests including serum creatinine(Cr), Alanine aminotransferase (ALT), and Aspartate aminotransferase (AST) were analyzed using chemistry analyzers: ABX Pentra 400(Horiba, USA), Dirui DR-7000D (DIRUI, Changchun, China) and HumaLyzer 3000 (HUMAN, Wiesbaden, Germany). Test for Hepatitis B surface antigen (HBsAg) was done using the rapid diagnostic tests Determine™ (Alere, Waltham, MA, USA), and Bio-Dignos HBsAg cassette(Nantong Diagnose, China); hepatitis C virus (HCV) antibody (anti-HCV) test was done using the rapid diagnostic test EGENS HCV Test cassette (Jiangsu, China), and SD BIOLINE HCV (Standard Diagnostics, Yongin-si, Republic of Korea). Human immunodeficiency virus (HIV) test was performed using the HIV 1/2 STAT-PAK® RDT (Chembio Diagnostics, Medford, NY, USA). Anti-nuclear antibody (ANA) was analyzed using the EliA Symphony assay (Phadia, Freiburg, Germany).

Abdominal ultrasonography was done by a radiologist using a D2.9MHz convex transducer (GE Logiq P6, Jiangsu, China), and 3.5 MHz convex transducer Aloka Flexus SSD-1100 (Aloka, Tokyo, Japan).

### Statistical analysis

Data were analyzed using STATA 13. For continuous data, a normality test was conducted using Shapiro-Wilk’s W test. For this purpose, level of significance of 0.05 was used. Parametric data were reported with mean and standard deviation and compared using student’s t-test. Non-parametric data were reported with median and interquartile range (IQR) and compared using the two-sample Wilcoxon rank-sum (Mann-Whitney) test. Proportions were compared using a chi-squared test or Fisher’s exact test, as appropriate. All *p* values calculated were two-sided, and the statistical significance threshold was <0.05. Survival experience of the patients was compared using Kaplan-Meier curve and log-rank test. The median time to clinical outcome and incidence rate ratio (IRR) were calculated. Bivariate Cox regression was performed to identify variables candidate for multivariable Cox regressions. Variables with p-value <0.25 in bivariate Cox regression were considered as candidates for multivariate cox-regression. After doing so, the interaction between independent variables was checked for collinearity before running multivariate cox regression. Finally, multivariable cox regression was performed. The hazard ratio was used to estimate the effect and p-value < 0.05 was considered to declare statistical significance.

### Outcome measurements

The primary outcome was in-hospital mortality. The following were also addressed: identifying etiology of CLD, in-hospital acute complications of CLD and 30-day mortality.

## Result

**Fig 1:**
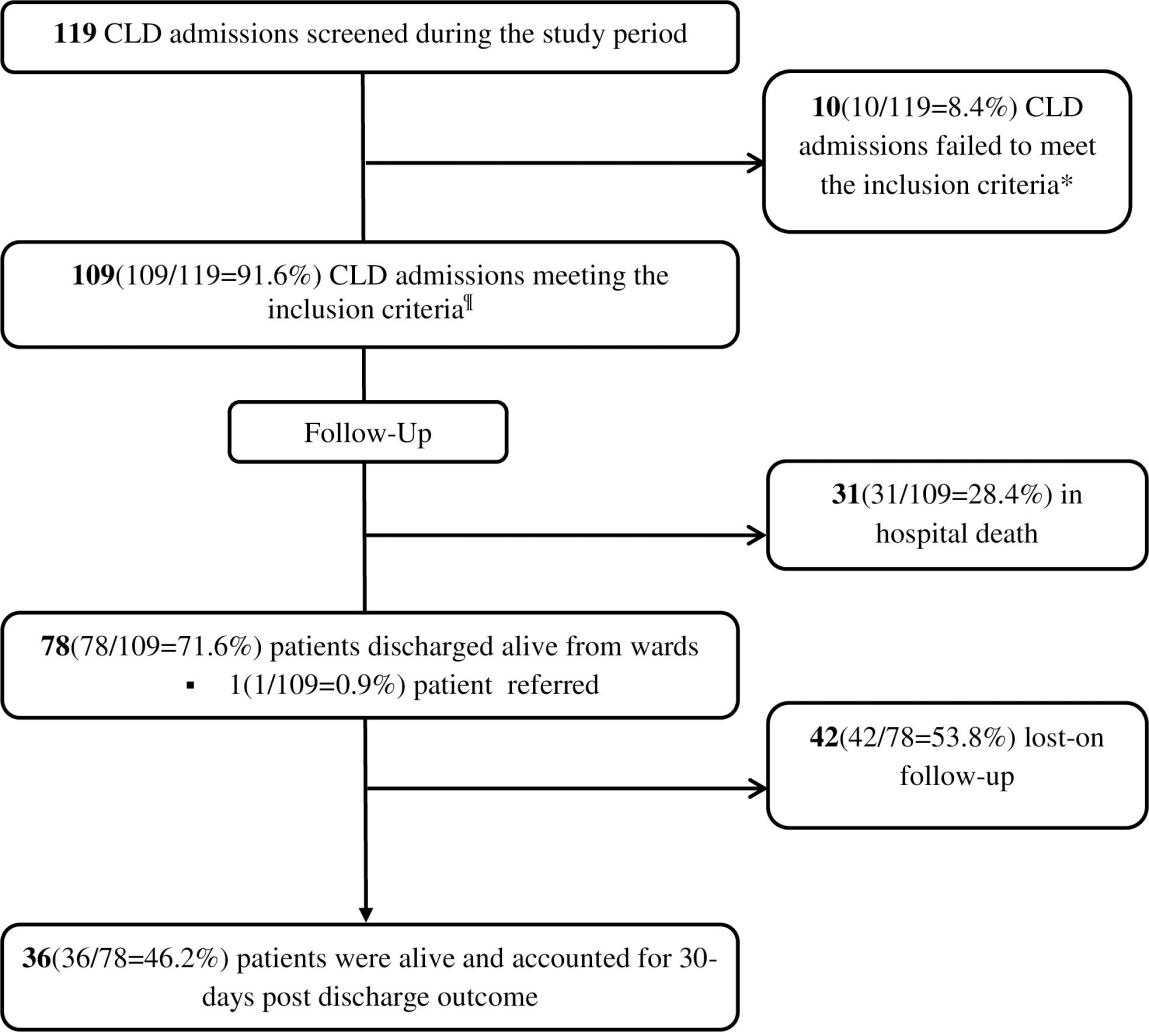
Consort flow chart of admitted CLD patients during the study period and those included in the study. *3 readmissions, 1 admission for another case other than CLD related complication(s), 4 individuals with a serologic test for viral hepatitis undone, and 2 pregnant women. ^¶^Hospitals contribution: JUMC = 61(61.0%), HFSUH = 27(27.0%), SPHMMC = 21(21.0%). **Acronyms:** JUMC-Jimma university medical center, HFSUH-Hiwot Fana Specialized university hospital, SPHMMC-Saint Paul hospital millennium medical college.

During the study period, there were a total of 119 CLD admissions to internal medicine wards of the selected hospitals, and 109 of these met the inclusion criteria. Eligible participants were followed for a median period of 9 days (IQR, 5–30 days). Consort flow chart of admitted CLD patients during the study period is depicted in **Fig 1.**

### Socio-demographic and behavioral characteristics

From the total of 109 eligible patients, 85(78%) of them were male. The median age of participants was 38(IQR, 30–48) years. Higher numbers of patients, 58(53.2%) were from rural areas. Religiously, 70 (64.2%) patients were Islam followers. A higher proportion of patients, 57(52.3%) had no formal education, and the majority of the patients, 78 (71.6%) were unemployed. Occupational status showed a significant association with hospital survival (*p =* **0.049).** In 72(66.1%) patients, average household income was less than 500 ETB (≈$18) per month. Herbal medicine use history was identified in 32(29.4%) patients. Regarding the history of social drug use habit, 52(47.7%), 39(35.8%) and 16(14.7%) of the participants reported khat chewing, alcohol consumption, and cigarette smoking history, respectively. In-hospital survivors were more likely to be khat chewers as compared to non-survivors (55.1% vs. 29%) (*p* ***=* 0.014**). Socio-demographic and behavioral characteristics are shown in **Table 1.**

**Table 1:** Socio-demographic and behavioral characteristics of CLD patients in relation to their association with in-hospital survival.

Characteristics		Total	Survivors (n = 78)	Non-survivors (n = 31)	*p-value*
Settings	JUMC	61(61.0%)	38(48.7%)	23(74.2%)	**0.004ǂ**
	HFSUH	27(27.0%)	26(33.3%)	1(3.2%)	
	SPHMMC	21(21.0%)	14(18%)	7(22.6%)	
Age, years, median(IQR)		38(30–48)	36.5(28–45)	40 (30–50)	0.134
Gender	Male	85(78%)	64(82.1%)	21(67.7%)	0.104
	Females	24(22.0%)	14(18%)	10(32.3%)	
Marital status					
	Never married	27(24.8%)	22(28.2%)	5(16.1%)	0.188
	Currently/formerly married	82(75.2%)	56(71.8%)	26(83.9%)	
Residence	Urban	51(46.8%)	33(42.3%)	18(58.1%)	0.137
	Rural	58(53.2%)	45(57.7%)	13(41.9%)	
Religion	Christianity	36(33%)	26(33.3%)	10(32.3%)	1.000
	Islam	70(64.2%)	50(64.1%)	20(64.5%)	
	Other(s)	3(2.8%)	2(2.6%)	1(3.2%)	
Educational status					
	Formal education	52(47.7%)	33(42.3%)	19(61.3%)	0.073
	No formal Education	57(52.3%)	45(57.7%)	12(38.7%)	
Occupation	Employed	31(28.4%)	18(23.1%)	13(41.9%)	**0.049ǂ**
	Unemployed	78(71.6%)	60(76.9%)	18(58.1%)	
Average household income(ETB)					
	<500(< ≈$18)	72(66.1%)	53(68%)	19(61.3%)	0.508
	≥500(≥ ≈$18)	37(33.9%)	25(32.1%)	12(38.7%)	
Herbal medication use history		32(29.4%)	25(32.1%)	7(22.6%)	0.327
khat chewing history		52(47.7%)	43(55.1%)	9(29%)	**0.014ǂ**
Cigarette smoking history		16(14.7%)	9(11.5%)	7(22.6%)	0.142
Alcohol consumption history		39(35.8%)	30(38.5%)	9(29%)	0.354
Clinical significance of alcohol consumption based on CAGE score					
	Clinically significant alcohol abuse	15(38.5%)	10(33.3%)	5(55.6%)	0.229
	Clinically non-significant alcohol use	24(61.5%)	20(66.7%)	4(44.4%)	

JUMC-Jimma university medical center, HFSUH-Hiwot Fana Specialized university hospital, SPHMMC-Saint Paul hospital millennium medical college, **IQR-Interquartile range.**

**ǂ** Statistically significant

### Baseline drug-related, clinical and laboratory characteristics

Abdominal ultrasound was done in 105(96.3%) patients. In-hospital survivors were found to be less likely to have ultrasound findings of heterogeneous echotexture than non-survivors (11.5% vs. 32.3%) (*p* ***=* 0.001**). Positive ultrasound impression of HCC was identified in 19(17.4%) patients. On serologic test, 39(35.8%) patients were HBsAg positive, and 12(11%) patients were anti-HCV positive. In this study, abdominal swelling, generalized body swelling, loss of consciousness, gastrointestinal bleeding, and abdominal pain were the major complaints of the patients presented in 47(43.1%), 20(18%), 14(13%), 11(10%), and 9(8%) patients, respectively. Yellowish discoloration of eyes was observed in 49 (44.9%) patients. Risk factors/Etiologies of CLD, ultrasound findings of the liver are depicted in **Table 2**.

**Table 2:** Risk factors/etiologies of CLD, and ultrasound findings of the liver in relation to their association with hospital outcome.

Characteristics	n, (%)	Survivor(n = 78)	Non-survivors (n = 31)	*p-value*
**Risk factors/Etiology of CLD** ^**a**^				
HBsAg +	39(35.8%)	30(75%)	10(25%)	0.544
Alcoholic	15(13.8%)	10(66.7%)	5(33.3%)	0.651
Anti-HCV+	12(11.0%)	9(69.2%)	4(30.8%)	0.843
Hepatic schistosomiasis	7(6.4%)	5(6.4%)	2(6.5%)	0.994
Unidentified	28(25.7%)	17(21.8%)	11(35.5%)	0.140
**Ultrasound findings**				
**Advanced liver fibrosis**				
Nodular liver surface	19(17.4%)	13(16.7%)	6(19.4%)	0.739
Coarse echotexture	16(14.7%)	12(15.4%)	4(12.9%)	0.741
Splenomegaly	37(33.9%)	29(37.2%)	8(25.8%)	0.258
**Liver fibrosis/inflammation**				
**Increased echogenicity**	21(19.3%)	13(16.7%)	8(25.8%)	0.275
**Heterogeneous echotexture**	19(17.4%)	9(11.5%)	10(32.3%)	**0.010ǂ**
**Mild uneven liver surface**	3(2.8%)	2(2.6%)	1(3.2%)	1.000
**Periportal fibrosis**	7(6.4%)	5(6.4%)	2(6.5%)	0.994
**Hepatocellular carcinoma**	19(17.4%)	11(14.1%)	8(25.8%)	0.146
**Ascites**	101(92.7%)	71(91%)	30(96.8%)	0.299
**Others**	10(9.2%)	9(11.5%)	1(3.2%)	0.175

^**a**^ HBsAg + and anti-HCV+, Autoimmune hepatitis, Wilson`s disease, biliary cirrhosis, Fatty liver

ǂ Statistically significant

The median number of CLD complications identified per individual at admission was 3(IQR, 2–5). Ascites was the most common complication at admission and observed in 101(92.7%) patients. Hepatic encephalopathy was the second most common complication and identified in 42(38.5%) patients at admission. In terms of the West Haven criteria of grading hepatic encephalopathy, 20(47.6%) patients had grade I. Gastrointestinal bleeding was diagnosed in 28(25.7%) patients at admission. Hepatorenal syndrome was diagnosed in 22(20.2%) patients, while 23(21.1%) patients were diagnosed with spontaneous bacterial peritonitis at presentation. Of the total patients diagnosed with spontaneous bacterial peritonitis, 21(91.3%) had neutrophil counts above 250/μL.

Chronic comorbidities were identified in 12(11%) patients. The identified comorbidities were: diabetes in seven (6.4%); chronic kidney disease and congestive heart failure each in three (2.8%), Human immunodeficiency virus/ Acquired immunodeficiency syndrome and peripheral vascular disease each in one (0.9%) patients.

At presentation, except two patients who were receiving antiviral medication (daily dose of Tenofovir 300mg); all patients were receiving supportive managements for CLD complication(s). Ceftriaxone was the cornerstone antibiotics initially prescribed for all patients diagnosed with spontaneous bacterial peritonitis. Diuretic(s) was/were used in 77(76.2%) ascitic patients, whereas therapeutic tap was performed in 45(44.5%) ascitic patients. Lactulose was the commonest agent (prescribed in 37) used for the treatment of patients with hepatic encephalopathy at admission. Combination treatment, lactulose with metronidazole, was used in 27 of the 42 patients admitted with hepatic encephalopathy. Due to resource limitation, endoscopic evaluation and band ligation was done for 8(7.3%) patients with gastrointestinal bleeding.

In this study, in-hospital survivors were found more likely to have lower white blood cell (WBC) count, mean corpuscular volume (MCV), mean corpuscular hemoglobin (MCH), platelet (PLT) count and total bilirubin (TB) level. Baseline drug-related, clinical and laboratory findings of the study participants are shown in **Table 3.**

**Table 3:** Baseline drug-related, clinical and laboratory findings of the study participants in relation to their association with hospital outcome.

Variables		Total (N = 109)	Survivors (n = 78)	Non-survivors (n = 31)	*p-value*
Drug-related factors					
Past diuretic(s) use history		35(32.1%)	27(34.6%)	8(25.8%)	0.374
Past propranolol use history		8(7.3%)	6(7.7%)	2(6.5%)	0.823
Diuretic(s) use during hospital stay		77(70.6%)	57.1(73.1%)	20(64.5%)	0.376
Therapeutic tap during hospital stay		45(41.3%)	28(35.9%)	17(54.8%)	0.070
Propranolol use during hospital stay		10(9.2%)	9(11.5%)	1(3.2%)	0.175
Lactulose use during hospital stay		37(33.9%)	25(32.1%)	12(38.7%)	0.508
Previously known CLD patients		34(31.2%)	50(64.1%)	25(80.7%)	0.093
Duration since diagnosis, months		7.85 (-120)	8.375 (0–96)	6.52(0–120)	0.094
Decompensated patients		108(99.1%)	77(98.7%)	31(100%)	0.716
Number of CLD complications diagnosed at admission		3(2–5)	3(2–5)	2(2–5)	0.428
Type of CLD complications at admission					
Ascites		101(92.7%)	71(91%)	30(96.8%)	0.299
Spontaneous Bacterial Peritonitis		23 (21.1%)	18(23.1%)	5(16.1%)	0.423
Gastrointestinal bleeding		28 (25.7%)	20(25.6%)	8(25.8%)	0.986
Hepatic encephalopathy		42 (38.5%)	27(34.6%)	15(48.4%)	0.183
Hepatic encephalopathy grade	I	20(47.6%)	12(44.4%)	8(53.3%)	0.838
	II	15(35.7%)	10(37%)	5(33.3%)	
	III	7(16.7%)	5(18.5%)	2(13.3%)	
Hepatorenal syndrome		22 (20.2%)	14(18%)	8(25.8%)	0.356
Hepatocellular carcinoma		19 (17.4%)	11(14.1%)	8(25.8%)	0.146
Comorbid Diabetes Mellitus		7(6.4%),	6(7.7%)	1(3.2%)	0.391
**Laboratory parameters**					
MAP, mmHg		82.33± 13.10	83.85± 13.69	78.53 ± 10.74	0.059
WBC,(10^9^/L)		7.2(4.5–11.4)	6.25(4.2–11.4)	9.1(7.2–12.7)	**0.006ǂ**
Hgb, g/dl		11(7.6–12.6)	11.1(8.2–12.5)	10.3(7.6–13)	0.824
MCV, fl		96.38 ± 90.84	82.74 ±13.01	130.69 ±166.05	**0.012ǂ**
MCH, pg		29.15(27–31)	28.75(27–30.3)	30.1(27.9–32.4)	**0.033ǂ**
MCHC, g/dl		33.6(32.2–35.1)	33.55 (32–34.8)	33.8 (33–36)	0.110
PLT(10^3^/L)		169 (95–248)	162.5(83–210)	178(154–392)	**0.014ǂ**
INR ^**a**^		1.67(1.29, 2.52)	1.63(1.31–2.46)	1.8(1.14–2.7)	0.671
Total bilirubin ^**b**^, mg/dl		2.09(1.03, 8.5)	1.85(0.9–5.69)	4.65(1.32–11.55)	**0.043ǂ**
ALT, u/l		45.5(29.3–72)	45.5(28.9–61.2)	47.2 (32.4–78)	0.197
AST, u/l		67 (42.0–143.5)	67(35–126.2)	90 (48.9–167.8)	0.088
ALP, u/l		256.5(169.1–348)	256.5(165–318.7)	256.5(169.1–437)	0.274
Scr, mg/dl		0.87(0.70–1.33)	0.89 (0.71–1.3)	0.85(0.55–1.7)	0.485
BUN, mg/dl		25.5 (20.0–39.72)	25.5(19.5–36.9)	25.5(21–48.9)	0.501

^**a**^ 54 missing values

^**b**^36 missing values

ALT- Alanine aminotransferase; AST-Aspartate aminotransferase; CLD-Chronic Liver Disease; HBsAg- Hepatitis B Virus Surface Antigen; MAP- Mean Arterial Pressure; MCV-Mean Corpuscular Volume; MCH-Mean Corpuscular Hemoglobin; MCHC-Mean Corpuscular Hemoglobin Concentration; PLT-Platelet; INR-International Normalized Ratio; WBC-White Blood Cell Count; Hgb-Hemoglobin; Scr-Serum creatinine; BUN-Blood Urea Nitrogen

**ǂ** Statistically significant

### Clinical outcomes

During hospital follow-up, the retention rate was 81.7%, and the median follow-up period was 4(IQR, 2.5–7) days for patients lost from the study over hospital stay, and 7(IQR,5–11) days for those followed-up until in-patient death or census date, respectively(*p* = **0.001**).

Seventeen (15.6%) patients developed new acute complications of CLD. More than one acute complications of CLD were identified in two patients. Nine (8.3%) of the complications were in the viral hepatitis group. The identified in-hospital acute complications of CLD were: acute gastrointestinal bleeding, hepatic encephalopathy and spontaneous bacterial peritonitis occurring in 7(8.6%), 6(9%) and 4(4.7%) patients, respectively. Hepatic encephalopathy was grade II in three of the six patients and grade I in the rest. The 30-day mortality was 38(34.9%); 18(16.5%) deaths were in the viral hepatitis group.

The overall analysis time at risk was 1806 days (927days in patients with viral hepatitis and 879 days in the non-viral hepatitis group). Incidence of mortality was not significantly different among patients with viral hepatitis and others, crude IRR 0.853 [0.426–1.699, *p* = 0.314]. In-hospital deaths were 31(28.4%); 13(11.9%) in the viral and 18(16.5%) in the non-viral hepatitis group. In-hospital overall analysis time at risk was 906 days; 482 days for the viral and 424 days for the non-viral hepatitis group (*p* = 0.43). Accordingly, the crude mortality IRR among viral to the non-viral hepatitis group was 0.635[95% CI, 0.286–1.371, *p = 0*.*108*]. The overall median survival time was 15(9–29) days; 29 days (13–29 days) for the patients with viral hepatitis and 12days for the non-viral hepatitis group(log-rank, *p* = **0.040**).

### Prognostic factors for survival

In the bivariate analysis, in-hospital mortality was found associated with Ultrasound finding of heterogeneous echotexture (*p =* **0.000**), being with unidentified risk factor/etiology (*p* = **0.046**), HE at admission (*p* = **0.011**), MCV (*p* **= <0.001**), Scr (*p* = **0.034**), and, total bilirubin (*p* = 0.001). In the multivariable, analysis, patients with hepatic encephalopathy at admission were found to have, approximately, 11 times increased risk of death as compared with patients without hepatic encephalopathy at admission. Similarly, patients in whom risk factor/etiology of CLD were not identified were at 5.8 times increased risk of death. The hazard of dying had also increased with an increase in total bilirubin (1.188 [95% CI, 1.0718–1.316, *p* **= 0.001**]) level. **Table 4**shows the cox regression survival analysis.

**Table 4:** Cox-regression survival analysis of patients hospitalized with Chronic liver disease (CLD) in relation to selected clinical findings as predictors.

Factors		CHR[95% CI ]	*P*-value	AHR[95% CI ]	*P*-value
khat chewing history					
	Yes	0.466[0.214–1.015]	**0.055**	0.299 [0.043–2.083]	0.223
	No	1		1	
Cigarette smoking history					
	Yes	1.985[0.842–4.677]	**0.117**	2.101[0.158–27.939]	0.574
	No	1		1	
Hepatic encephalopathy			**0.011**		
	Yes	2.708 [1.252–5.859]		11.361[2.391–53.987]	**0.002ǂ**
	No	1		1	
HBsAg+		0.570[0.265–1.230]	0.152	1.395[0.1502–12.959]	0.770
Unidentified risk factor/etiology		2.113[1.012–4.437]	**0.046**	5.803[1.380–24.405]	**0.016ǂ**
	Yes	0.577 [0.078 4.261]	**0.59**		
	No	1			
MAP, mmHg		0.973 [0.940–1.007]	**0.117**	0.962[0.888–1.042]	0.346
WBC, (10^9^/L)		1.036 [0.986–1.089]	**0.161**	1.056[0.941–1.186]	0.351
Hgb, g/dl		1.010 [0.897–1.137]	0.868		
MCV, fl		1.005 [1.00–1.007]	**<0.001**	1.005[0.999–1.011]	0.095
Total bilirubin, mg/dl		1.084[1.034–1.138]	**0.001**	1.188[1.0719–1.316]	**0.001ǂ**
ALT, u/l,		1.004 [0.998–1.010]	**0.240**	0.992 [0.981–1.004]	0.194
Scr, mg/dl		1.233[1.016–1.495]	**0.034**	0.686[0.459–1.025]	0.066
Nodular liver surface		0.475[0.178–1.266]	**0.136**	3.699[0.724–18.895]	0.116
Splenomegaly		0 .532[0.236–1.2]	**0.128**	2.236[0.540–9.267]	0.267
Heterogeneous echotexture		5.906[2.484–14.044]	**0.000**	2.645[0.403–17.358]	0.311
In-hospital acute Complication of CLD					
	Yes	1.607[0.752–3.432]	**0.221**	1.886 [0.413–8.613]	0.413
	No	1			

ALT- Alanine aminotransferase; CLD-Chronic Liver Disease; HBsAg- Hepatitis B Virus Surface Antigen; MAP- Mean Arterial Pressure, MCV-Mean Corpuscular Volume; WBC-White Blood Cell Count; Scr-Serum Creatinine

**ǂ** Statistically significant

**Fig 2:**
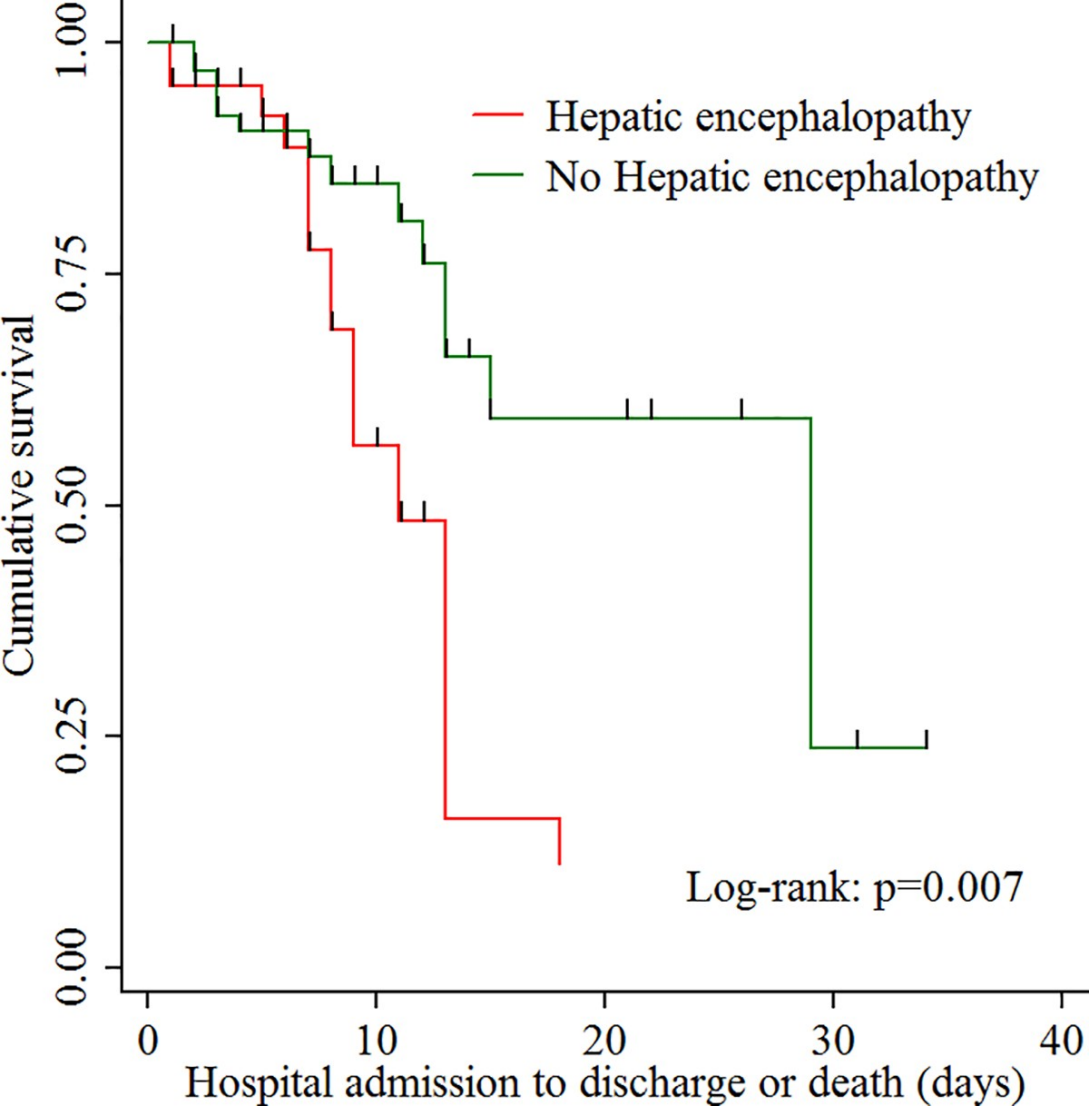
This is a Kaplan-Meier survival analysis of patients admitted with Chronic Liver disease (CLD) across the course of hospital stay stratified by the presence of hepatic encephalopathy. As the time of hospital stay increased, the survival of patients with hepatic encephalopathy (red line) was less than those without hepatic encephalopathy (green line).

**Fig 3:**
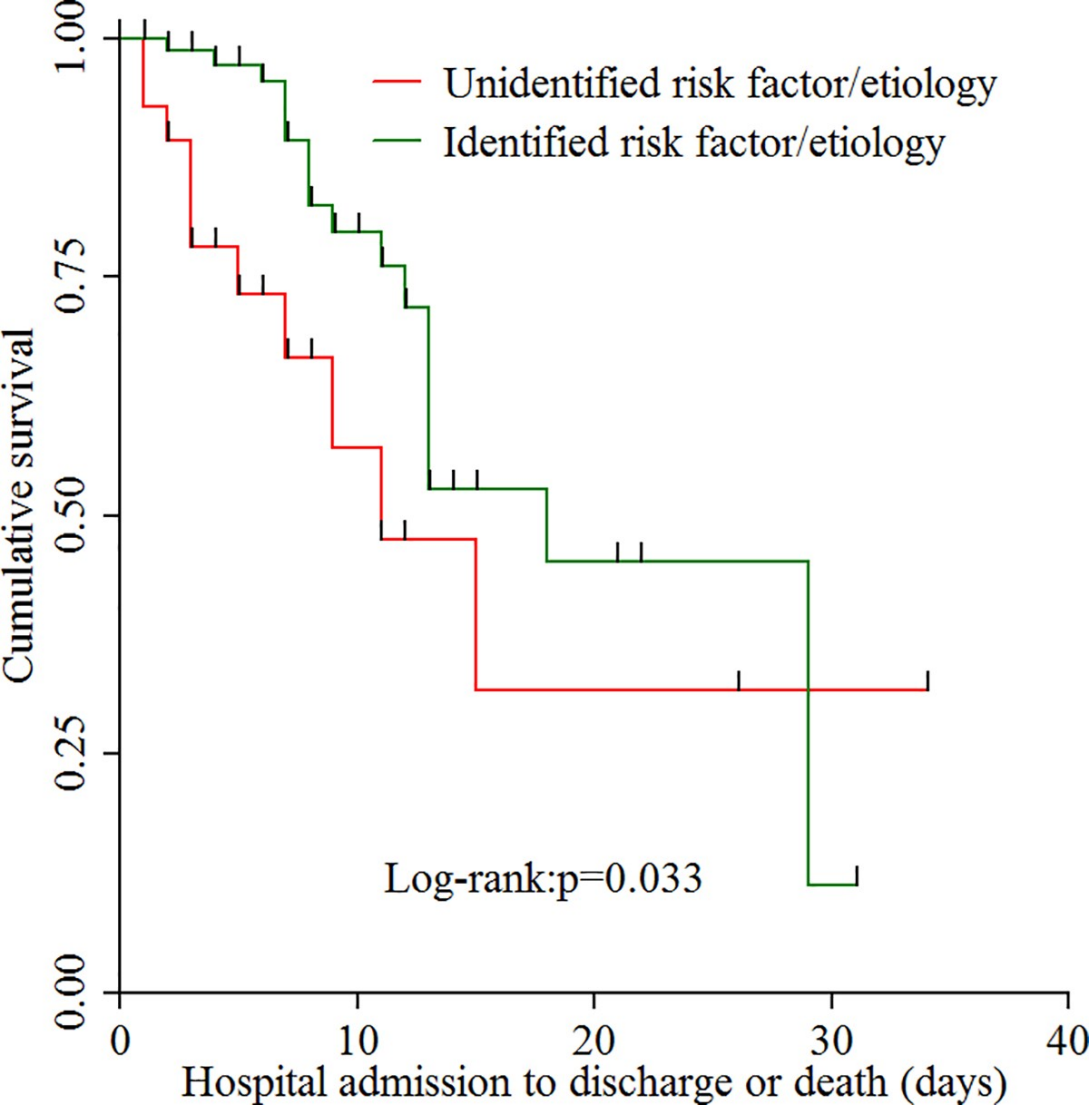
This is a Kaplan-Meier survival analysis of patients admitted with Chronic Liver disease (CLD) across the course of hospital stay stratified by presence of identification of risk factor/etiology of CLD. As the time of hospital stay increased, the survival of patients with unidentified risk factor/etiology (red line) was less than those with identified risk factor/etiology (green line), but after approximately 30-days the patients with unidentified risk factor/etiology were better survived.

As a complement to the findings in **Table 4**, Kaplan-Meier survival analysis of mortality were done taking the period from hospital admission to discharge or death as time frame; and hepatic encephalopathy at admission and unidentified risk factor/etiology as independent factors. As the time of hospital stay increased, the survival of patients with hepatic encephalopathy was less than those without hepatic encephalopathy (Log-Rank: *p* = **0.007**) (Fig 2). Similarly, the survival of patients with unidentified risk factor/etiology was lower than patients with identified risk factor/etiology as the duration of hospital stay increased (Log-Rank: *p* = **0.033**) (Fig 3).

## Discussion

This study aimed to assess baseline characteristics and short-term clinical outcomes of CLD patients treated at three selected specialized hospitals in Ethiopia. Accordingly, in this cohort of patients with short-term follow-up, we described the identified risk factors/etiologies. HBV is reported to be the main etiology of CLD in developing countries[26]. Similar to this, chronic HBV (35.8%) infection was identified as the most common etiology of CLD in this study. This result was consistent with other studies conducted in Ethiopia [14,15,27]. Studies from Newzealand [28] and India [29] have also reported chronic HBV as predominant etiology of CLD. Inconsistent to our finding, in a study conducted in Australia by Fagan et al. [8], alcohol was reported as the commonest etiology of CLD. Two studies conducted in Greece by Samonakis et al. [3] and Giannousis et al. [30], reported HCV as the primary etiology of CLD. This inconsistency could be due to a geographical difference in etiology of CLD[31,32]. Although, more than one-third of the patients were chronic HBV positive, in the present study, only a very limited number of patients received antiviral treatment. Besides, no patient received immunoprophylaxis.

The 30-days mortality was 38(34.9%). Of these, 31(28.4%) deaths were in-hospital. The similar in-hospital mortality rate was also reported in a study from Colombia[33]. But, higher in-hospital mortality as compared to our finding was reported by two other studies [14,34]. On the other hand, lower in-hospital mortality was documented from Argentinean and Moroccan studies[35,36]. These discrepancies could be partly due to the difference in settings included. Moreover, the current study included patients admitted to medical wards only, while others were conducted among patients admitted to the critical care unit or else included patients admitted to both ICU and internal medicine ward. Besides, a potential difference in baseline characteristics could also exist between patients included in these studies. Furthermore, late presentation, unavailability of specific therapies and advanced hepatology centers in Ethiopia could also have contributed to the discrepancy seen.

In the present study, the hazard of in-hospital mortality was found to increase by more than eleven-fold among patients with hepatic encephalopathy at admission as compared to those not diagnosed with hepatic encephalopathy In line with this, studies have shown an increase in the risk of mortality among patients with hepatic encephalopathy at presentation[36,37]. Hepatic encephalopathy is associated with poor outcomes with an overall decline in liver function, increased risk of in-hospital and short-term mortality[38,39,40].

Furthermore, in our study, etiology of CLD was not identified in more than a quarter of the patients. This finding is lower than the reports of a study done in Ethiopia by half [15]. Those CLD patients with unidentified etiology were found to have a higher risk of dying as compared to those with identified etiology of CLD.

An increase in the level of bilirubin was also found to increase the risk of death. An increase in total bilirubin has also been found as an independent predictor of mortality in studies from the United Kingdom [34], and France[41].

Finally, as nothing is without limitation, our study has limitations too. The small sample size employed; the large number of lost to follow-up of the participants; lack of some diagnostic facilities like advanced imaging, molecular viral assays, endoscopy; lack of standardization between laboratories, and participant selection bias were also the limitations of the present study.

## Conclusion

Approximately, one death was observed for four admissions with CLD. A further increase in mortality was also noticed with short-term follow-up after discharge. Given that more than one third the patients had HBV-infection, access to antiviral drugs could help improve the prognosis of patients with end-stage liver disease in Ethiopia, as well as prevent the development of cirrhosis if initiated earlier. Furthermore, prevention and early identification of modifiable risk factors, early diagnosis and management of hepatic encephalopathy are also warranted.

## Supporting information

S1 Dataset Data collection tool. (ZIP)

S2 Dataset Short_term Outcomes of CLD. (ZIP)

## Abbreviations

ALD: Alcoholic Liver Disease
CLD: Chronic Liver Disease
GBD: Global Burden of Disease Study
HBsAg: Hepatitis B Surface Antigen
HBV: Hepatitis B Virus
HCC: Hepatocellular Carcinoma
HCV: Hepatitis C Virus
HE: Hepatic Encephalopathy
HFSUH: Hiwot Fana Specialized University Hospital
JUMC: Jimma University Medical Center
SPHMMC: St. Paul’s Hospital Millennium Medical College
