# Supplementary material for: Short-term clinical outcomes of patients admitted with chronic liver disease to selected teaching hospitals in Ethiopia
Source: PLoS One. 2019 Aug 30;14(8):e0221806. doi: 10.1371/journal.pone.0221806 (PMC6716656; doi:10.1371/journal.pone.0221806)
Supplement: S1 Dataset — (ZIP) [file pone.0221806.s001.zip › Amharic_English version data collection tool.docx]

የተከበሩ፡ ይህ መረጃ መሰብሰቢያ የተዘጋጀዉ በሶስት የጤና መዓከላት ተኝቶ ታከሚያ ክፍል ዉስጥ ተኚተዉ በሚታከሙ ህሙማን መካከል ስር የሰደደ ጉበት በሽታ የህክምና ዉጤት ምን እንደሚመስል ለማወቅ ነዉ። ለዚህም መሳካት የታካሚ ተሳትፎ አስፈላጊ ይሆናል፡፡ ማንኛዉም ተሳታፊ ከመሳተፉ በፊት፥ የተሳታፊዎች መረጃ ቅጽ እና የስምምነት ሰነዱን በማንበብ በመሳተፍ ከተስማማ የስምምነት ሰነዱ ላይ መስማማቱን እንዲያረጋግጥ በትህትና እንጠይቃለን።

ከወዲዉ ስለትብብርዎ እናመሰግናለን

Dear all, this data collection tool is designed to assess short-term clinical outcomes of patients admitted with chronic liver disease to three specialized teaching hospitals in Ethiopia. For realizing this study, kind participation of chronic liver disease patient is required. Before any participant engages in this study, they are recommended to read **patient information sheet** and **give their written informed consent on the paper ready for this purpose,** if they agree to participate.

**Thank you in advance.**

| 1. **Socio-demographic** **characteristics of the patient ስልክ ቁጥር. የመደዎያ ቀን:** | | | | | | |
| --- | --- | --- | --- | --- | --- | --- |
| **የካርድ ቁጥር:___________**   1. **እድሜ:**____ 2. **ጾታ:**  ወንድ  ሴት 3. **ሆስፒታል የገቡበት ቀን:**__________ 4. **የመኖሪያ ቦታ:**  ከተማ  ገጠር 5. **ታካሚዉ የጉበት ህመም እንዳለበት የተነገረዉ/ራት**（ቀን/ወር/ዓም？_______________ 6. **የትዳር ሁኔታ:**  ያላገባ  ያገባ  የፈታ  የትዳር ጓደኛዉ በሞት የተለየ 7. **ሃይማኖት:**  ክርስቲያን ሙስሊም  ሌላ 8. **የትምህርት ደረጃ:**  ኢመደበኛ  1ኛ ደረጃ  2ኛ ደረጃ  ከፍተኛ ደረጃ ማንበብና መጻፍ የማይችል 9. **ሥራ:** የመንግስት ሥራተኛ ወታደር የጤና ባለሙያ ጡረተኛ የግል ስራ የቀን ሰራተኛ ስራ የለለዉ 10. **ወራዊ ገቢ**(ብር):___________ 11. **ትንባሆ ያጨሳሉ?**  ቀድሞ አጨስ ነበር አዎ አሁንም ድረስ አጨሳለዉ በፍጹም 12. **ጫት ይጠቀማሉ?** ቀድሞ እጠቀም ነበር  አዎ አሁንም ድረስ እጠቀማለዉ በፍጹም 13. **በሓላዊ መድሃኒት ተጠቅመዉ ያዉቃሉ?**  አዎ  አይ | | | | 1. **አልኮል መጠጥ ይጠጣሉ?**  ቀድሞ እጠጣ ነበር አዎ አሁንም ድረስ እጠጣሳለዉ በፍጹም 2. **አልኮል መጠጥ ተጠቅመዉ የሚያዉቁ ከሆነ፣ እባኮን የሚከተሉትን ጥያቀዎች ይመልሱ（መልስዎ አዎ ከሆነ=1፣አይ ከሆነ=0）**Screening of alcohol abuse using CAGE criteria. If the answer is `yes` write 1 if `no` write 0) 3. አልኮል መጠጣት መተዉ አለብኝ ብለው አስበው ያዉቃሉ？（Have you ever felt you should **cut** down on your drinking? ） 4. አልኮል በመጠጣቶት ምክኒያት ሰዉ በትችት አበሳጭቶት ያዉቃል？   （Have people **annoyed** you by criticizing your drinking? ）   1. አልኮል በመጠጣቶት ምክኒያት መጥፎ/የጥፋተኝነት ስሜት ተሰምትዎት ያዉቃል（Have you ever felt bad or **guilty** about your drinking?） 2. ጥዋት ከእንቅልፎ እንደነቁ ለመነቃቃት/ከአዳሪ መጥፎ ስሜት ለመላቀቅ ብለው አልኮል ጠጥተዉ ያዉቃሉ？（Have you ever had a drink first thing in the morning to steady your nerves or to get rid of a hangover(**eye-opener**)） | | |
| 1. **Baseline disease characteristics of the patient** | | | | | | |
| 1. **ስር ለሰደደ የጉበት ህምም መንስዔዉ ምንድነዉ？እባኮን ከሚከተሉት ዝርዝሮች ዉስጥ የትኛዉ እንደሆነ ሳጥኑን ✓ምልክት በማድረግ ያሳዩ？（**What is the etiology of chronic liver disease in this patient? | | | | | | |
| አልኮል（Alcoholism） | HBV | HCV | NAFLD | | Biliary cirrhosis |  Wilson’s disease |
| Hepatic schistosomiasis | Cryptogenic | Unknown | AIH | |  Other(s), _________________ | |
| 1. **በዋናነት ወደ ሆስፒታል ኢዲመጡ ያደረጎት ምንድነዉ?** | | | | | | |

| 1. **ባለፉት ሶስት ወራት የወሰዱት/እየወሰዱ ያለ መድሃኒት አለ？** አዎ  አይ | | | | |
| --- | --- | --- | --- | --- |
| 1. **ለ20ኛ ጥያቄ መልሶት አዎ ከሆነ：የመድሃኒቱ ስም ምን ይባላል** | | | | |
| Lasix | Metronidazole | |  | |
| Spironolactone | Norfloxacin | |  | |
| Propranolol | Ceftriaxone | |  | |
| Paracetamol or NSAIDs | Enalapril or other ACEIs | |  | |
| Proton pump inhibitor like Omeprazole | Anti-TB medications | |  | |
| 1. **ስር ከሰደደ የጉበት ህምም ጋር ተያይዘዉ ከሚመጡ ተጓዳኝ ህመሞች ዉስጥ ታካሚዉ ወደ ሆስፒታል እንደገባ የተገኘበት ካለ፥እባኮን ከሚከተሉት ዝርዝሮች ዉስጥ የትኛዉ እንደሆነ ሳጥኑን ✓ምልክት በማድረግ ያሳዩ？**（If the patient is diagnosed with CLD Ccomplication(s) at admission, please tick ✓in the box corresponding to the complication(s)） | | | | |
| **ተጓዳኝ ህመሞች** （Complication(s) ） | | **የምርመራ አይነት（**Diagnosis method used） | | **የተደረገለት ህክምና**（Management(s) including paracentesis） |
|  Ascites | |  | |  |
| Variceal bleeding/gastrointestinal bleeding | | Clinical Endoscopy | |  |
| Hepatic encephalopathy  Grade: I II III IV | | | |  |
| Spontaneous bacterial peritonitis | | lab.(PMN≥250 cells/m^3^)  Clinical | |  |
| Hepatocellular carcinoma | | US | |  |
| Others (please specify) | |  | |  |

1. **ታካሚዉ ላይ የታዩ ምልክቶችን ይዘርዝሩ** (Signs and symptoms at admission)
2. **የላብራቶሪ ምርመራዎች（Laboratory tests）**

| **COMPLETE BLOOD COUNT(CBC)** | **LIVER FUNCTION TEST (LFT)** | **ASCITIC FLUID ANALYSIS** |
| --- | --- | --- |
| - RBC count: - WBC count: - Platelet count: - Hemoglobin: - Hematocrit: - Neutrophil count: - Lymphocyte count: - Monocyte count: - Basophil count: - Eosinophils: - MCV: - MCH: - MCHC: | - ALT: - AST: - ALP: | PMN cells count: |
|  | **COAGULATION PROFILE** | **RENAL FUNCTION TEST(RFT)** |
|  | - PT: - INR: - aPTT: | - Scr - BUN |
|  | **SERUM BILIRUBIN** | **BLOOD GLUCOSE(mg/dl)** |
|  | - Direct: - Indirect: - Total: | - FBS - RBS |
| **Blood pressure at admission (mm Hg):** | **SERUM ALBUMIN(g/dL):** | **SEROLOGIC TESTS** |
| **BP1:___________**  **BP2:___________**  **BP3:___________** |  | - HBsAg: Positive Negative - Anti-HCV: Positive Negative - Anti-nuclear antibody (ANA) Reactive Non-reactive - HIV(PIHCT) Reactive Non-reactive |

1. **የአልትራሳዉንድ ዉጤት**（Ultrasound findings (Please tick ✓ in the corresponding box based on the ultrasound finding)）

- Ascites
- Smooth liver surface
- Mild uneven liver surface
- Nodular liver surface
- Heterogeneous echotexture
- Coarse echotexture
- Hepatic steatosis
- Periportal fibrosis
- Other(s), please specify briefly

1. **ስር ከሰደደ ጉበት ህመም እና ተጓዳኝ ህመሞች ዉጭ ሌላ በሺታ ካለ（Comorbidity questions）**

| - **ታካሚዉ** **ስር ከሰደደ የጉበት ህመም እና ተጓዳኝ ህመሞች ዉጭ ሌላ በሺታ አለበት？（**Does the patient have chronic comorbidity currently? ） አዎ  አይ |
| --- |
| - **ስር ከሰደደ ጉበት ህመም እና ተጓዳኝ ህመሞች ዉጭ ሌላ በሺታ ካለ እባኮን ዪዘርዝሩ （**If the patient have chronic comorbidity currently, please list the comorbidities） |

1. **የታካሚዉ የሆስፒታል እና ከሆስፒታል ከወጣ ሰላሳ ቀን ሲሞላዉ ያለዉ ዉጤት**（In-hospital, and 30-days post discharge outcomes）

| - **ታካሚዉ ሆስፒታል ሲገባ ያልነበረበት ስር የሰደደ የጉበት ህመም ተጓዳኝ ህመም/ሞች ታይቶበታል？（**Did the patient developed acute complication(s) of CLD while in-hospital? አዎን አይ | |
| --- | --- |
| - **ስር የሰደደ የጉበት ህመም ተጓዳኝ ህመም/ሞች ታይቶበት ከሆነ ፥ እባኮን ከሚከተሉት ዘርዝሮች ዉስጥ የትኛዉ እንደሆነ ሳጥኑን ✓ምልክት በማድረግ ያሳዩ（** If the patient developed acute complication(s) of CLD while in-hospital, please tick ✓in the box corresponding to the complication(s) | |
|  | **የምርመራ አይነት(**What was the method of diagnosis used?) |
| Variceal bleeding | Clinical Endoscopy |
| Hepatic encephalopathy  Grade:  I  II  III  IV |  |
| Spontaneous bacterial peritonitis |  Lab.(PMN≥250 cells/m^3^) Clinical |
|  Hepatorenal syndrome | - Scr: |
| Other(s),please specify ___________ |  |
| **ታካሚዉ የሆስፒታል ቆዪታ ዉጤት ምንድነዉ？**（What is the final in-hospital outcome of the patient? | **የሆስፒታል ዉጤቱ የተከሰተበት ቀን/ወር （**In-hospital outcome occurrence…DD/MM） |
| ተሺሎት ተሸኘ（Patient improved and discharged）  ወድ ለላ የጤና ማእከል ተላከ（Referred/worsened）   ህክምናዉን ሳይጨርስ በራሱ ፍቃድ አቋረጠ（Patient Left against medical advice）   ታካሚዊ ህክምናዉን አቋርጦ ጠፋ（patient lost on follow-up）  ህይወቱ አለፈ（Patient passed away） |  |
| **ታካሚዉ ከሆስፒታል ከወጣ በኋላ የሰላሳኛ ቀን ዉጤት ምንድነዉ？**（What had happened to the patient at 30-days of hospital discharge? ）(only for patients improved and discharged) | **ታካሚዉ ከሆስፒታል ከወጣ በኋላ የሰላሳኛ ቀን ዉጤት የተከሰተበት ቀን/ወር** （Thirty-day of hospital discharge outcome occurrence ..DD/MM) |
| ህይወቱ አለፈ（Patient passed away）  ታካሚዉን በድጋሚ ሆስፒታል ገባ （Patient readmitted）   ታካሚዉን በህይወት አለ（Patient survived）  ታካሚዉን ማግኘት አልተቻለም（Patient lost to follow-up） |  |

ስለትብብርዎ እናመሰግናለን（Thanks for you cooperation）
