# Supplementary material for: Short-term clinical outcomes of patients admitted with chronic liver disease to selected teaching hospitals in Ethiopia
Source: PLoS One. 2019 Aug 30;14(8):e0221806. doi: 10.1371/journal.pone.0221806 (PMC6716656; doi:10.1371/journal.pone.0221806)
Supplement: S1 Dataset — (ZIP) [file pone.0221806.s001.zip › English version data collection tool.docx]

Dear all, this data collection tool is designed to assess short-term clinical outcomes of patients admitted with chronic liver disease to three specialized teaching hospitals in Ethiopia. For realizing this study, kind participation of chronic liver disease patient is required. Before any participant engages in this study, they are recommended to read **patient information sheet** and **give their written informed consent on the paper ready for this purpose,** if they agree to participate.

**Thank you in advance.**

| 1. **Socio-demographic** **characteristics of the patient Tel.** | | | | | | |
| --- | --- | --- | --- | --- | --- | --- |
| **Card number:___________**   1. **Age:**____ 2. **Sex:**  M  F 3. **Admission date:**__________ 4. **Residence:**  Urban  Rural 5. Duration of Chronic liver disease? _______________ 6. **Marital status:**  single  married  divorced  Widowed 7. **Religion:**  Christian Muslim others 8. **Educational status:**  Informal Primary Secondary Tertiary unable to read and write 9. **Educational status** Civil servant Military Health worker Retired Self-employed Daily labor No work 10. **Average monthly income (birr)**:___________ 11. **Cigarette Smoking history**: Yes No 12. **Khat Chewing history**: Yes : No 13. **Herbal medication use history:** Yes No | | | | 1. **Alcohol use history:** **Active alcoholic** **Inactive alcoholic** **Denied** 2. The following four questions are only for patients who have alcohol use history **(**Screening of alcohol abuse using CAGE criteria. If the answer is `yes` write 1 if `no` write 0) 3. Have you ever felt you should **cut** down on your drinking? 4. Have people **annoyed** you by criticizing your drinking? 5. Have you ever felt bad or **guilty** about your drinking? 6. Have you ever had a drink first thing in the morning to steady your nerves or to get rid of a hangover(**eye-opener**) | | |
| 1. **Clinical characteristics of the patient** | | | | | | |
| 1. What is the etiology of chronic liver disease in this patient? | | | | | | |
| Alcoholism | HBV | HCV | NAFLD | | Biliary cirrhosis |  Wilson’s disease |
| Hepatic schistosomiasis | Cryptogenic | Unknown | AIH | |  Other(s), _________________ | |
| 1. **Main presenting complaint(s) of the patient** | | | | | | |

| 1. **Past medication history (medication use history in the past 03 months): **Yes No | | | |
| --- | --- | --- | --- |
| 1. **If there is medication use history in the past 03 months, what medication? Please tick✓if it corresponds to the list below.** | | | |
| Lasix | | others | |
| Spironolactone | |  | |
| Propranolol | |  | |
| 1. If the patient is diagnosed with CLD Complication(s) at admission, please tick ✓in the box corresponding to the complication(s) | | | |
| Complication(s) | Diagnosis method used | | Management(s) including paracentesis |
|  Ascites |  | |  |
| Variceal bleeding/gastrointestinal bleeding | Clinical Endoscopy | |  |
| Hepatic encephalopathy  Grade: I II III IV | | |  |
| Spontaneous bacterial peritonitis | lab.(PMN≥250 cells/m^3^)  Clinical | |  |
| Hepatocellular carcinoma | US | |  |
| Others (please specify) |  | |  |

1. Signs and symptoms at admission
2. **Laboratory tests**

| **COMPLETE BLOOD COUNT(CBC)** | **LIVER FUNCTION TEST (LFT)** | **ASCITIC FLUID ANALYSIS** |
| --- | --- | --- |
| - RBC count: - WBC count: - Platelet count: - Hemoglobin: - Hematocrit: - Neutrophil count: - Lymphocyte count: - Monocyte count: - Basophil count: - Eosinophils: - MCV: - MCH: - MCHC: | - ALT: - AST: - ALP: | PMN cells count: |
|  | **COAGULATION PROFILE** | **RENAL FUNCTION TEST(RFT)** |
|  | - PT: - INR: - aPTT: | - Scr - BUN |
|  | **SERUM BILIRUBIN** | **BLOOD GLUCOSE(mg/dl)** |
|  | - Direct: - Indirect: - Total: | - FBS - RBS |
| **Blood pressure at admission (mm Hg):** | **SERUM ALBUMIN(g/dL):** | **SEROLOGIC TESTS** |
| **BP1:___________**  **BP2:___________**  **BP3:___________** |  | - HBsAg: Positive Negative - Anti-HCV: Positive Negative - Anti-nuclear antibody (ANA) Reactive Non-reactive - HIV(PIHCT) Reactive Non-reactive |

1. Ultrasound findings (Please tick ✓ in the corresponding box based on the ultrasound finding)

- Ascites
- Smooth liver surface
- Mild uneven liver surface
- Nodular liver surface
- Heterogeneous echotexture
- Coarse echotexture
- Hepatic steatosis
- Periportal fibrosis
- Other(s), please specify briefly

1. **Comorbidity questions**

| - Does the patient have chronic comorbidity currently? ） Yes  No |
| --- |
| - If the patient have chronic comorbidity currently, please list the comorbidities） |

1. In-hospital, and 30-days post discharge outcomes

| - Did the patient developed acute complication(s) of CLD while in-hospital? Yes No | |
| --- | --- |
| - If the patient developed acute complication(s) of CLD while in-hospital, please tick ✓in the box corresponding to the complication(s) | |
|  | What was the method of diagnosis used?) |
| Variceal bleeding | Clinical Endoscopy |
| Hepatic encephalopathy  Grade:  I  II  III  IV |  |
| Spontaneous bacterial peritonitis |  Lab.(PMN≥250 cells/m^3^) Clinical |
|  Hepatorenal syndrome | - Scr: |
| Other(s),please specify ___________ |  |
| What is the final in-hospital outcome of the patient? | In-hospital outcome occurrence…DD/MM |
| Patient improved and discharged  Referred/worsened   Patient Left against medical advice   patient lost on follow-up  Patient passed away |  |
| What had happened to the patient at 30-days of hospital discharge? (only for patients improved and discharged) | Thirty-day of hospital discharge outcome occurrence…DD/MM |
| Patient passed away  Patient readmitted   Patient survived  Patient lost to follow-up |  |

Thanks for your cooperation.
